# Changes in the Global Diet Quality Score, Weight, and Waist Circumference in Mexican Women

**DOI:** 10.1093/jn/nxab171

**Published:** 2021-10-23

**Authors:** Erick Angulo, Dalia Stern, Analí Castellanos-Gutiérrez, Adriana Monge, Martín Lajous, Sabri Bromage, Teresa T Fung, Yanping Li, Shilpa N Bhupathiraju, Megan Deitchler, Walter C Willett, Carolina Batis

**Affiliations:** Health and Nutrition Research Center, National Institute of Public Health, Cuernavaca, Mexico; CONACYT—Center for Research on Population Health, National Institute of Public Health, Cuernavaca, Mexico; Health and Nutrition Research Center, National Institute of Public Health, Cuernavaca, Mexico; Center for Research on Population Health, National Institute of Public Health, Cuernavaca, Mexico; Center for Research on Population Health, National Institute of Public Health, Cuernavaca, Mexico; Department of Global Health and Population, Harvard TH Chan School of Public Health, Boston, MA, USA; Department of Nutrition, Harvard TH Chan School of Public Health, Boston, MA, USA; Department of Nutrition, Harvard TH Chan School of Public Health, Boston, MA, USA; Department of Nutrition, Harvard TH Chan School of Public Health, Boston, MA, USA; Department of Nutrition, Harvard TH Chan School of Public Health, Boston, MA, USA; Intake—Center for Dietary Assessment, FHI Solutions, Washington, DC, USA; Department of Nutrition, Harvard TH Chan School of Public Health, Boston, MA, USA; CONACYT—Health and Nutrition Research Center, National Institute of Public Health, Cuernavaca, Mexico

**Keywords:** diet quality, diet quality metrics, dietary diversity, GDQS, weight change, waist circumference change, Mexican women

## Abstract

**Background:**

Evidence on concurrent changes in overall diet quality and weight and waist circumference in women of reproductive age from low- and middle-income countries is limited.

**Objectives:**

We examined the associations of changes in the Global Diet Quality Score (GDQS) and each GDQS food group with concurrent weight and waist circumference change in Mexican women.

**Methods:**

We followed prospectively 8967 nonpregnant nonlactating women aged 25–49 y in the Mexican Teachers’ Cohort between 2006 and 2008. We assessed diet using an FFQ of the previous year and anthropometric measures were self-reported. Regression models were used to examine 2-y changes in the GDQS and each food group (servings/d) with weight and waist circumference changes within the same period, adjusting for demographic and lifestyle factors.

**Results:**

Compared with those with little change in the GDQS (−2 to 2 points), women with the largest increase in the GDQS (>5 points) had less weight (β: −0.81 kg/2 y; 95% CI: −1.11, −0.51 kg/2 y) and waist circumference gain (β: −1.05 cm/2 y; 95% CI: −1.62, −0.48 cm/2 y); likewise, women with the largest decrease in the GDQS (<−5 points) had more weight (β: 0.36 kg/2 y; 95% CI: 0.06, 0.66 kg/2 y) and waist circumference gain (β: 0.71 cm/2 y; 95% CI: 0.09, 1.32 cm/2 y). Increased intake of dark green leafy vegetables, cruciferous vegetables, deep orange vegetables, citrus fruits, and fish and shellfish was associated with less weight gain. In addition, deep orange vegetables, low fat and high fat dairy, whole grains, and fish were associated with less waist circumference gain within the 2-y period.

**Conclusions:**

Improvements in diet quality over a 2-y period reflected by an increase in the GDQS and changes in consumption of specific components of the GDQS were associated with less weight and waist circumference gain in Mexican women.

## Introduction

Excessive weight gain is an important determinant for the development of noncommunicable diseases (NCDs) such as diabetes mellitus (1), cardiovascular diseases (2), and various types of cancers (3). In Mexico, the prevalence of obesity in adults has increased from 25.1% in 2000 to 35.6% in 2018 and the increase has been larger in women than in men (from 30.1% to 39.3% among women, and from 19.7% to 30.6% among men) (4). Several risk factors have been identified that contribute to the development of obesity. Evidence from epidemiological studies has shown that the most important environmental factors that contribute to weight gain are poor diet quality, physical inactivity, and an obesogenic built environment (5). Mexico has faced changes in dietary patterns, from a traditional diet characterized by maize foods, fruits, and vegetables to a Western diet characterized by sugar-sweetened beverages (SSBs), white bread, fast food, sweets, and snacks (6–8). However, evidence in Mexico on the association between dietary quality and weight or waist circumference change is still limited.

To assess diet quality in a population, a priori dietary patterns or indices are commonly used (9). These metrics measure the adherence to specified dietary patterns or dietary guidelines, or reflect the risk gradient for major diet-related chronic diseases (10). A number of longitudinal studies have shown a favorable association between overall diet quality and body weight. For instance, in the Nurses’ Health Study II, an increase in the Alternate Healthy Eating Index-2010 (AHEI-2010) was associated with less weight gain (11); likewise, women from Australia who improved their diet quality according to the Australian Recommended Food Score (ARFS) gained significantly less weight (12). Until now, several diet quality indices have been developed, but overall they are designed for a particular geographic area or population and a particular nutritional need (e.g., nutrient adequacy or chronic diseases) (13). Furthermore, most diet quality metrics have been developed for high-income countries (14).

Given the importance of diet quality as one of the largest threats to global public health and the usefulness of diet quality metrics to capture this exposure, a global metric (inclusive of low- and middle-income countries) capable of capturing dietary risk in relation to nutrient adequacy as well as chronic disease and body weight is needed. To fill this gap, the Global Dietary Quality Score (GDQS) is proposed. In this study, we focused on 1 of the outcomes the GDQS aims to capture. Our aim was to evaluate the association between the GDQS and weight and waist circumference change in Mexican women of reproductive age. To better understand the performance of the GDQS in our outcome of interest and in this population, we also evaluated the independent association of each of the food groups included in the GDQS. Finally, we compared the performance of the GDQS with 2 other established dietary indices, the AHEI-2010 and Minimum Dietary Diversity for Women (MDD-W) indicator.

## Methods

### Population

The Mexican Teachers’ Cohort (MTC) is a prospective study of >115,000 female teachers aged 25 y or older. The cohort was initiated in 2006 among women from 2 states (Jalisco and Veracruz) and in 2008 these women completed their first follow-up; at the same time, the cohort was expanded to include women from 10 additional states across Mexico. The average enrollment rate was 64%, and the median age at enrollment was 44 y (15). In each wave (2006, 2008, and 2011), participants responded to questionnaires on sociodemographic characteristics, reproductive history, lifestyle, and medical conditions. A diet questionnaire was only included in 2006 and 2008, therefore for this analysis we only included women from Jalisco and Veracruz that participated in both 2006 and 2008. Of the 27,979 female teachers that were enrolled in 2006 from Veracruz and Jalisco, 19,130 completed a follow-up questionnaire in 2008. We included 13,419 women who in both 2006 and 2008 were 49 y old or younger, not pregnant, and not lactating; and who in 2006 did not report diabetes, cancer, or heart disease. We excluded women with inadequate dietary information in either 2006 or 2008 [energy intake <500 or >3500 kcal/d, missing response to ≥70 items on the dietary questionnaire, or any missing data on the staple grains section (because of their important contribution to energy intake); *n* = 3382]. We also excluded women with missing height and/or weight (*n* = 1070), thus our analytical sample was 8967 women in the weight change analysis. For waist circumference, 1379 women had missing values, thus our analytical sample was 7588 women in the waist circumference change analysis.

### Diet assessment and diet quality score computation

Diet was measured using a 139-item semiquantitative FFQ derived from a previously validated 116-item FFQ in Mexico City. Correlation coefficients in the previous validation analysis for total energy, carbohydrate, protein, and total fat intakes between the FFQ and four 4-d 24-h recalls were 0.52, 0.57, 0.32, and 0.63, respectively (16). Informed by National Nutrition Surveys, 23 food items were added to the MTC FFQ to capture regional differences and secular changes in food consumption, and to include other foods such as low-calorie options and different food varieties (e.g., lean fish or fatty fish).

For each food item, women were asked to specify how often, on average, they had consumed a specified commonly used unit or portion size of the food or beverage over the previous year. Ten multiple-choice frequencies of consumption were possible: ≥6/d, 4–5/d, 2–3/d, 1/d, 5–6/wk, 2–4/wk, 1/wk, 2–3/mo, ≤1/mo, and never. We converted food frequency responses of each food item to servings per day and then to grams per day using specified portion sizes. Energy and nutrient intakes were estimated using a food composition table derived from a database developed by the National Institute of Nutrition and Medical Sciences in Mexico and the USDA nutrient database (17).

The GDQS is a food-based dietary quality score modified from the Prime Diet Quality Score (18) to assess nutrient adequacy and study the association between chronic diseases in a global context. The GDQS includes 25 food groups in total: 16 healthy food groups (citrus fruits, deep orange fruits, other fruits, dark green leafy vegetables, cruciferous vegetables, deep orange vegetables, other vegetables, deep orange tubers, legumes, nuts and seeds, whole grains, liquid oils, fish and shellfish, poultry and game meat, low fat dairy, and eggs), 2 unhealthy groups when consumed in excess (high fat dairy and red meat), and 7 unhealthy food groups (processed meat, refined grains and baked goods, sweets and ice cream, SSBs, juice, white roots and tubers, and purchased deep fried foods) (19).

We classified 125 food items from the MTC FFQ into 23 food groups because the MTC FFQ does not ask for any food that could be included in the groups for liquid oils or deep orange tubers. All of these food groups include 3 categories of consumption quantity which are used in the scoring of the metric, except for high fat dairy which includes 4 categories. For the healthy food groups, the higher the intake the higher the score, whereas for unhealthy food groups, the lower the intake the higher the score. In the case of high fat dairy and red meat, the score is amount-dependent (i.e., moderate consumption gets the highest score) owing to the food's potential contribution to nutrient intake at modest amounts of consumption, while also recognizing its potential contribution to NCD risk associated with higher amounts of consumption. Two submetrics can be estimated from the GDQS. The positive submetric, the GDQS+, is obtained by summing only the point values for healthy food groups, whereas the negative submetric, the GDQS−, is obtained by summing only the point values for unhealthy food groups (red meat and high fat dairy included). The original GDQS has a range from 0 to 49, but in our study, the range was 0 to 46.5 because of the 2 food groups that were not captured in our FFQ. We also estimated the servings per day of each food group included in the GDQS to evaluate their contribution to weight and waist circumference change.

We compared the performance of the GDQS with 2 other indices: the AHEI-2010 and the MDD-W. The AHEI-2010 includes foods and nutrients that have been shown to lower or raise the risk of major chronic disease (20). The AHEI-2010 awards points for higher consumption of vegetables, fruits, whole grains, nuts and legumes, long-chain n–3 FAs, and n–6 polyunsaturated fat; for lower consumption of SSBs and fruit juices, red/processed meat, sodium, and *trans* fat; and for moderate alcohol consumption. Each component has a range of 0–10 points, with a maximum overall score of 110 points (11). The MDD-W was developed by the Food and Nutrition Technical Assistance Project and the FAO of the UN. It is based on 10 food groups that predicted micronutrient intake adequacy in women of reproductive age from low-income countries (21). The food groups included are grains, white roots, and tubers; legumes; nuts and seeds; dairy; meat, poultry, and fish; eggs; dark green leafy vegetables; vitamin A–rich fruits and vegetables; other vegetables; and other fruits. To compute the MDD-W score, we used an intake of ≥1 serving/d as the cutoff to assign 1 point for each food group, otherwise we gave a score of 0. The MDD-W total score ranges from 0 to 10 points (18).

Diet quality scores were computed for each individual in 2006 and 2008. However, to calculate diet quality scores in 2006, we imputed the egg consumption from 2008 to 2006, because this was not asked in the 2006 FFQ. Evidence suggests that egg consumption does not change much over time (22).

### Weight and waist circumference assessment

On the 2006 and 2008 questionnaires, participants self-reported height (cm) and weight (kg) and were provided a plastic measuring tape and instructions to assess their waist circumference (cm). A previous study evaluated the reproducibility and validity of self-reported anthropometry in a subset of 3413 participants. Standardized technician measurements were well correlated with self-reported weight (*r* = 0.92), height (*r* = 0.86), and waist circumference (*r* = 0.78) (23). We calculated changes in weight and waist circumference by subtracting self-reported measures in 2008 from those in 2006.

### Assessment of covariates

The 2006 and 2008 questionnaires included participant characteristics such as age, marital status, education, household assets, and type of health insurance (public, private, and other); lifestyle habits such as smoking status, alcohol consumption, and physical activity; and any recent physician-diagnosed disease. Physical activity was assessed through a self-report of the average hours spent each week over the prior year doing any of the following activities using 8 response categories ranging from 0 to >10 h/wk: walking, moderate physical activity at work, moderate recreational physical activity, vigorous physical activity at work, and vigorous recreational physical activity. To quantify the intensity of physical activities, each specific activity was assigned a metabolic equivalent task (MET) score based on a compendium of physical activities (24). Total physical activity was defined as the sum of specific MET-hours per week for each reported activity and we categorized it into tertiles (low, medium, and high). The correlation between this physical activity questionnaire and the International Physical Activity Questionnaire was 0.64 for moderate and vigorous physical activity (Pearson correlation coefficient: 0.64; 95% CI: 0.54, 0.97; Intraclass correlation coefficient: 0.77; 95% CI: 0.64, 0.86) (unpublished results).

We used the number of household assets, including car, telephone, cell phone, microwave, vacuum, computer, and internet, to create a socioeconomic position (SEP) score (25) and categorized it into tertiles (lowest, medium, and highest).

### Statistical analysis

Changes in diet quality scores (GDQS, GDQS+, GDQS−, AHEI-2010, and MDD-W), food groups, weight, and waist circumference were calculated by subtracting 2008 values from 2006 values. Participants with changes <1^st^ percentile or >99^th^ percentile of the distribution were assigned the value at the 1^st^ or 99^th^ percentile, accordingly, to minimize the influence of extreme values. Participants with missing data in categorical covariates were assigned into a missing category and were not excluded from the analysis; this was the case for 2% of the sample for health insurance, 5% for alcohol drinking, 6% for smoking, and 12% for education.

We categorized 2-y changes in GDQS as largest decrease (<−5 points), small decrease (−5 to <−2 points), little change (−2 to 2 points), small increase (>2 to 5 points), and largest increase (>5 points). These cutoffs were chosen because these were close to the quintiles’ cutoffs, but are easier to compare across populations and studies; for instance, these cutoffs were also used in the Nurses’ Health Study (26). We estimated means and proportions of the GDQS, weight, waist circumference, and covariates by the categorical change in GDQS. We ran linear regression models to examine the associations between categorical 2-y change in the GDQS and continuous 2-y change of weight and waist circumference. Our reference group was little change (−2 to 2 points) in the GDQS. Similarly, we ran linear regression models to examine the association between change in the GDQS+ and GDQS− submetrics, which were categorized in quintiles using quintile 3 (little change) as the reference group. In this case, we used the exact cutoffs of the quintiles because the distribution was very specific to our population and to each submetric. A test for linear trend across categories was performed by assigning the median value to each category and modeling it as a single continuous variable. We also explored the linear association between change in consumption of each food group of the GDQS (servings/d) and 2-y change in weight and waist circumference using a linear regression model, where all food groups were mutually adjusted.

To compare the performance of the GDQS with the AHEI-2010 and the MDD-W, we examined the association with weight and waist circumference change per 1-SD increase in each diet quality index over the 2-y period. We modeled a 1-SD increase in each diet quality indicator at a time, and in a model with the GDQS plus each of the other diet quality indicators in the same regression model to test for the difference in weight and waist circumference change between the 2 diet quality scores with the Wald test. Finally, given the large prevalence of overweight and obesity and to understand if our results differed by baseline BMI we tested the interaction between changes in diet quality indices as continuous variables and baseline BMI (in kg/m^2^; <25 compared with ≥25), with changes in weight and waist circumference as the dependent variables.

All models were adjusted for the following potential confounders: baseline age (continuous), state (Jalisco, Veracruz), change in energy intake (continuous), baseline GDQS (continuous), 2006 and 2008 physical activity (tertiles: low, middle, high; we were unable to calculate changes in recreational physical activity because of differences in the 2006 assessment compared with the 2008 assessment), baseline marital status (single, living together, married, separated, widow), baseline education (none, high school or less, undergraduate degree, graduate degree or above), baseline household assets (tertiles: lowest, medium, highest), and health insurance (public, private, other). We also adjusted for baseline BMI (<25, 25–30, and ≥30) because it can be associated with changes in both weight and diet quality (e.g., women with overweight or obesity may have improved their diet as a treatment of weight loss at the initial stage). We also adjusted for change in smoking status [baseline past smoker, starters (change from never or former to current smoker), quitters (change from current to former smoker), nonsmokers (stayed former or never smoker), smokers (stayed smoker)] and change in alcohol drinking [baseline nondrinker, starters (change from nondrinker to drinker), quitters (change from drinker to nondrinker), nondrinkers (stayed nondrinker), drinkers (stayed drinker)]. All analyses were conducted using SAS version 7.1 (SAS Institute).

## Results

The mean ± SD age at baseline was 41.4 ± 3.1 y, the mean ± SD BMI was 26.8 ± 4.3, and 63% of the sample was overweight or obese. Mean ± SD weight and waist circumference change over 2 y of follow-up was 1.1 ± 4.0 kg and 0.99 ± 7.1 cm, respectively. Mean ± SD GDQS change was an increase of 0.18 ± 4.0 points. The proportion of teachers who were smokers and alcohol drinkers in 2006 and stayed as such in 2008 was 5.7% and 53.4%, respectively (data not shown). Compared with women with the largest decrease in GDQS, women with the largest increase in GDQS were more likely to be obese and less likely to be in the highest SEP and physical activity categories at baseline and were more likely to remain nonsmokers and nondrinkers in 2008. The GDQS was positively associated with energy intake; for instance, women with higher GDQS at baseline had higher energy intake, and women with the largest increase in GDQS also had an increase in energy intake. Therefore, subsequent analyses were adjusted for energy intake (**Table 1**).

**TABLE 1:** Sample characteristics by change in GDQS: Mexican Teachers’ Cohort, 2006–2008^1^

	Largest decrease (<−5) (*n* = 865)	Small decrease (−5 to <−2) (*n* = 1709)	Little change (−2 to 2) (*n* = 3661)	Small increase (>2 to 5) (*n* = 1722)	Largest increase (>5) (*n* = 1010)
Age, baseline, y	41.4 ± 3.1	41.3 ± 3.1	41.4 ± 3.2	41.3 ± 3.1	41.2 ± 3.2
GDQS, baseline	26.7 ± 3.7	24.9 ± 3.9	22.9 ± 4.0	21.1 ± 3.8	18.9 ± 3.8
GDQS, 2-y change	−7.0 ± 1.3	−3.5 ± 0.8	0.0 ± 1.2	3.5 ± 0.8	7.1 ± 1.6
Weight, baseline, kg	65.8 ± 11.3	66.2 ± 11.5	66.2 ± 11.6	66.5 ± 12.0	66.7 ± 11.6
Weight, 2-y change, kg	1.5 ± 4.2	1.4 ± 3.9	1.2 ± 4.0	0.8 ± 4.1	0.5 ± 4.1
WC, baseline, cm	85.4 ± 11.0	86.2 ± 10.5	86.0 ± 10.3	86.5 ± 10.4	87.3 ± 10.7
WC, 2-y change, cm	1.5 ± 7.4	1.3 ± 7.2	1.1 ± 6.9	0.5 ± 7.2	0.2 ± 7.2
Energy intake, baseline, kcal	2038 ± 630	1915 ± 620	1837 ± 639	1739 ± 607	1647 ± 619
Energy intake, 2-y change, kcal	−402 ± 676	−206 ± 609	−60 ± 583	86 ± 591	288 ± 664
Baseline BMI, kg/m^2^					
<25	38.6	38.3	37.2	36.7	34.5
≥25	61.3	62.0	62.6	63.2	65.3
Smoking					
Baseline past smoker	15.0	13.5	12.2	12.1	11.9
Starter	3.8	1.8	2.4	2.4	2.8
Quitter	1.9	1.8	2.0	1.8	2.1
Stayed smoker	6.7	5.5	5.7	5.7	4.9
Stayed nonsmoker	72.3	77.2	77.5	77.6	78.1
Alcohol drinking					
Baseline nondrinker	2.0	1.4	1.3	1.3	1.6
Starter	7.5	7.1	7.1	6.9	7.2
Quitter	9.2	11.3	9.8	10.1	11.3
Stayed drinker	54.5	53.1	54.5	52.0	51.9
Stayed nondrinker	26.5	27.0	27.0	29.5	27.8
Physical activity					
Low, baseline	35.5	37.7	37.2	39.5	39.3
Middle, baseline	27.9	28.2	28.0	27.8	30.4
High, baseline	36.4	33.9	34.6	32.6	30.2
Low, 2008	38.0	33.8	34.4	29.9	31.8
Middle, 2008	31.8	33.7	33.4	32.5	32.9
High, 2008	30.0	32.4	32.1	37.5	35.2
Household assets, baseline					
Lowest	38.3	37.2	37.8	41.5	45.7
Medium	35.9	37.1	35.6	33.4	32.9
Highest	25.6	25.6	26.4	25.0	21.2
Education, 2008					
None	2.2	1.1	2.1	1.4	1.6
High school or less	7.7	7.0	7.8	7.2	7.5
Undergraduate degree	79.0	79.1	81.1	82.6	80.7
Graduate degree or above	11.0	12.6	8.9	8.6	10.0
Marital status, baseline					
Single	14.2	15.7	16.4	14.4	15.0
Living together	9.4	9.5	10.0	10.5	11.2
Married	66.0	64.3	63.1	64.6	63.4
Separated	8.9	9.2	8.6	9.1	8.6
Widow	1.4	1.1	1.6	1.2	1.5
Health insurance, baseline					
Public	76.0	77.8	78.3	79.6	79.2
Private	20.8	19.4	19.0	17.6	17.8
Other	3.1	2.7	2.6	2.7	2.9

1 *n* = 8967. Values are means ± SDs or percentages. Household assets comprised phone, car, computer, vacuum cleaner, microwave oven, cell phone, and internet. Missing data: smoking, *n* = 569; alcohol drinking, *n* = 456; physical activity 2006, *n* = 33; physical activity 2008, *n* = 95; education, *n* = 1139; marital status, *n* = 78; health insurance, *n* = 204. GDQS, Global Diet Quality Score; WC, waist circumference.

After adjusting for potential confounders, compared with women with little change in their GDQS, women with the largest increase in their GDQS had less weight gain (β: −0.81 kg/2 y; 95% CI: −1.11, −0.51 kg/2 y), whereas women with the largest decrease in their GDQS had more weight gain (β: 0.50 kg/2 y; 95% CI: 0.19, 0.81 kg/2 y) within the 2-y period. Likewise, we observed an association between an increase in GDQS and waist circumference change. Compared with women with little change in their GDQS, those with the largest increase had less waist circumference gain (β: −1.05 cm/2 y; 95% CI: −1.62, −0.48 cm/2 y) and those with the largest decrease had more waist circumference gain (β: 0.71 cm/2 y; 95% CI: 0.09, 1.32 cm/2 y) within the 2-y period (**Table 2**).

**TABLE 2:** Association between change in GDQS and weight and waist circumference change within a 2-y period: Mexican Teachers’ Cohort^1^

	Largest decrease	Small decrease	Little change	Small increase	Largest increase	*P*-trend^2^
Total GDQS change	<−5	−5 to <−2	−2 to 2	>2 to 5	>5	
Weight change, kg						
Age- and state-adjusted	0.36 (0.06, 0.66)	0.24 (0.01, 0.47)	Reference	−0.39 (−0.62, −0.15)	−0.70 (−0.98, −0.41)	<0.0001
Multivariable-adjusted^3^	0.50 (0.19, 0.81)	0.33 (0.09, 0.57)	Reference	−0.43 (−0.67, −0.20)	−0.81 (−1.11, −0.51)	<0.0001
Waist circumference change, cm						
Age- and state-adjusted	0.54 (0.04, 1.12)	0.24 (−0.19, 0.69)	Reference	−0.49 (−0.93, −0.05)	−0.99 (−1.53, −0.45)	<0.0001
Multivariable-adjusted^3^	0.71 (0.09, 1.32)	0.32 (−0.12, 0.77)	Reference	−0.49 (−0.94, −0.04)	−1.05 (−1.62, −0.48)	<0.0001
GDQS+ score change	<−2.7	−2.0 to <−0.2	0 to 1.7	>2.0 to 4.0	>4.2	
Weight change, kg						
Age- and state-adjusted^4^	0.22 (−0.04, 0.49)	0.11 (−0.15, 0.37)	Reference	−0.19 (−0.45, 0.07)	−0.50 (−0.76, −0.23)	<0.0001
Multivariable-adjusted^3^^,^^4^	0.22 (−0.04, 0.50)	0.10 (−0.16, 0.36)	Reference	−0.21 (−0.47, 0.05)	−0.52 (−0.79, −0.24)	<0.0001
Waist circumference change, cm						
Age- and state-adjusted^4^	0.30 (−0.19, 0.81)	0.10 (−0.41, 0.61)	Reference	−0.21 (−0.72, 0.29)	−0.81 (−1.32, −0.30)	0.0002
Multivariable-adjusted^3^^,^^4^	0.32 (−0.19, 0.84)	0.10 (−0.41, 0.61)	Reference	−0.22 (−0.73, 0.28)	−0.79 (−1.32, −0.27)	<0.0001
GDQS− score change	<−2.0	−1.0	0	1.0	>2.0	
Weight change, kg						
Age- and state-adjusted^4^	0.36 (0.10, 0.62)	0.10 (−0.17, 0.39)	Reference	−0.25 (−0.53, 0.02)	−0.32 (−0.59, −0.06)	<0.0001
Multivariable-adjusted^3^^,^^4^	0.36 (0.10, 0.62)	0.14 (−0.14, 0.42)	Reference	−0.20 (−0.48, 0.07)	−0.25 (−0.51, 0.01)	<0.0001
Waist circumference change, cm						
Age- and state-adjusted^4^	0.95 (0.46, 1.45)	0.78 (0.24, 1.32)	Reference	0.45 (−0.08, 0.99)	−0.14 (−0.64, 0.35)	0.0143
Multivariable-adjusted^3^^,^^4^	0.98 (0.47, 1.48)	0.80 (0.25, 1.34)	Reference	0.49 (−0.04, 1.03)	−0.07 (−0.57, 0.43)	0.0082

1 Values are β coefficients (95% CIs). *n* = 8967 women were included in the weight change analysis; *n* = 7588 women were included in the waist circumference analysis. GDQS+ and GDQS− scores were categorized in quintiles. GDQS, Global Diet Quality Score; GDQS+, Global Diet Quality Score positive submetric; GDQS−, Global Diet Quality Score negative submetric.

2 Medians were fitted in a multivariate model to estimate *P*-trend.

3 Values were adjusted for baseline age (continuous); change in energy (continuous); state (Jalisco, Veracruz); baseline GDQS (continuous); 2006 and 2008 physical activity (low, medium, high); baseline marital status (single, living together, married, separated, widow); baseline education (none, high school or less, undergraduate degree, graduate degree or above); baseline household assets (lowest, medium, highest); baseline health insurance (public, private, other); baseline BMI (<25, 25–29.9, >30 kg/m^2^); and changes in smoking status (baseline past smokers, stayed nonsmokers, stayed smokers, quitters, starters) and alcohol consumption (baseline nondrinkers, stayed nondrinkers, stayed drinkers, quitters, starters).

4 Mutually adjusted for subscores.

We examined the associations between change in the GDQS submetric scores and weight and waist circumference change. Compared with women with little change, the largest increase in GDQS+ score was associated with less weight (β: −0.52 kg/2 y; 95% CI: −0.79, −0.24 kg/2 y) and waist circumference (β: −0.79 cm/2 y; 95% CI: −1.32, −0.27 cm/2 y) gain, whereas the largest decrease in the GDQS+ was not associated with weight and waist circumference change. Furthermore, the largest decrease in GDQS− score was associated with more weight (β: 0.36 kg/2 y; 95% CI: 0.10, 0.62 kg/2 y) and waist circumference (β: 0.98 cm/2 y; 95% CI: 0.47, 1.48 cm/2 y) gain, whereas the largest increase in the GDQS− was not associated with weight and waist circumference change within the 2-y period (Table 2).

**FIGURE 1:**
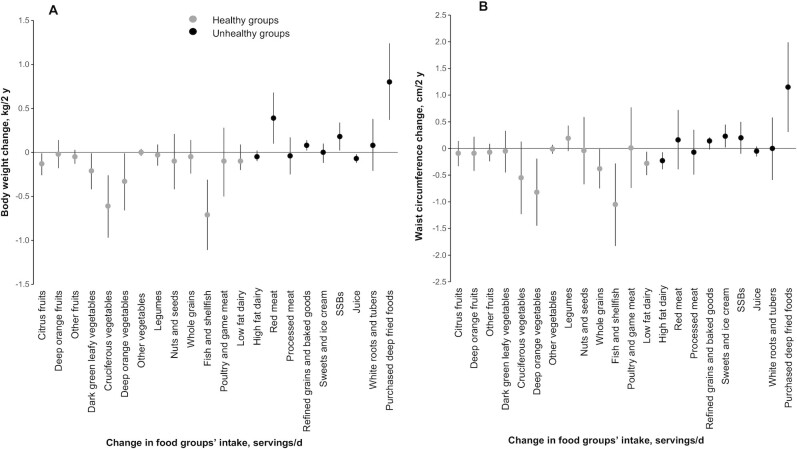
Relation between change in consumption of food groups (servings/d) and weight (A) and waist circumference (B) change within a 2-y period: Mexican Teachers’ Cohort. Values were adjusted for baseline age (continuous); change in energy (continuous); state (Jalisco, Veracruz); 2006 and 2008 physical activity (low, medium, high); marital status (single, living together, married, separated, widow); education (none, high school or less, undergraduate degree, graduate degree or above); baseline household assets (lowest, medium, highest); health insurance (public, private, other); baseline BMI (<25, 25–29.9, >30 kg/m^2^); and changes in smoking status (baseline past smokers, stayed nonsmokers, stayed smokers, quitters, starters) and alcohol consumption (baseline nondrinkers, stayed nondrinkers, stayed drinkers, quitters, starters). The model was mutually adjusted for other food groups. SSB, sugar-sweetened beverage.

With regards to the healthy and unhealthy food groups included in the GDQS, a 1-serving increase per day of healthy foods was inversely associated with weight gain for citrus fruits (−0.13 kg), dark green leafy vegetables (−0.21 kg), cruciferous vegetables (−0.61 kg), deep orange vegetables (−0.33 kg), and fish and shellfish (−0.71 kg) (*P* < 0.05). A 1-serving increase per day of unhealthy foods was positively associated with weight gain for red meat (0.40 kg), refined grains (0.08 kg), SSBs (0.18 kg), and purchased deep fried foods (0.80 kg) (*P* < 0.05) (**Figure 1**A). In the case of waist circumference, we found inverse associations with increased consumption of deep orange vegetables (−0.83 cm), fish and shellfish (−1.05 cm), whole grains (−0.38 cm), and low fat dairy (−0.28 cm), and positive associations with sweets and ice cream (0.23 cm) and purchased deep fried foods (1.14 cm) (*P* < 0.05) (Figure 1B).

When comparing the strength of association with weight and waist circumference change of the GDQS, the AHEI-2010, and the MDD-W (unadjusted by each other), we found that 1-SD increases in all 3 scores were associated with less weight gain (GDQS: β: −0.37 kg/2 y; 95% CI: −0.47, −0.27 kg/2 y; AHEI-2010: β: −0.33 kg/2 y; 95% CI: −0.44, −0.22 kg/2 y; MDD-W: β: −0.26 kg/2 y; 95% CI: −0.37, −0.14 kg/2 y) and waist circumference gain (GDQS: β: −0.52 cm/2 y; 95% CI: −0.71, −0.33 cm/2 y; AHEI-2010: β: −0.24 cm/2 y; 95% CI: −0.45, −0.03 cm/2 y; MDD-W: −0.42 cm/2 y; 95% CI: −0.63, −0.20 cm/2 y). When adjusting by each other the GDQS and AHEI-2010 were significantly associated with less weight, whereas the MDD-W was not associated. For waist circumference gain, only the GDQS was significantly associated. Comparing the strength of the coefficients, the GDQS and MDD-W coefficients were statistically significantly different (Wald test *P* = 0.008) for weight gain and the GDQS and AHEI-2010 (Wald test *P* = 0.006) for waist circumference (**Table 3**).

**TABLE 3:** Association between change in diet quality indices (1-SD increase) and weight and waist circumference change within a 2-y period: Mexican Teachers’ Cohort^1^

	GDQS	AHEI-2010	MDD-W	Wald test
Weight change, kg				
Unadjusted for another score	−0.37 (−0.47, −0.27)	−0.33 (−0.44, −0.22)	−0.26 (−0.37, −0.14)	
Adjusted for GDQS	−0.28 (−0.40, −0.16)	−0.18 (−0.31, −0.05)	—	0.383
Adjusted for GDQS	−0.36 (−0.47, −0.24)	—	−0.08 (−0.21, 0.04)	0.008
Waist circumference change, cm				
Unadjusted for another score	−0.52 (−0.71, −0.33)	−0.24 (−0.45, −0.03)	−0.42 (−0.63, −0.20)	
Adjusted for GDQS	−0.54 (−0.78, −0.31)	0.04 (−0.20, 0.30)	—	0.006
Adjusted for GDQS	−0.43 (−0.65, −0.21)	—	−0.22 (−0.47, 0.01)	0.300

1 Values are β coefficients (95% CIs). Values were adjusted for baseline age (continuous); change in energy (continuous); baseline scores (continuous); state (Jalisco, Veracruz); 2006 and 2008 physical activity (low, medium, high); marital status (single, living together, married, separated, widow); education (none, high school or less, undergraduate degree, graduate degree or above); baseline household assets (lowest, medium, highest); health insurance (public, private, other); baseline BMI (<25, 25–29.9, >30 kg/m^2^); and changes in smoking status (baseline past smokers, stayed nonsmokers, stayed smokers, quitters, starters) and alcohol consumption (baseline nondrinkers, stayed nondrinkers, stayed drinkers, quitters, starters). AHEI-2010, Alternate Healthy Eating Index-2010; GDQS, Global Diet Quality Score; MDD-W, Minimum Dietary Diversity for Women.

We also tested if there was an interaction between the diet quality indices (GDQS, AHEI-2010, and MDD-W) and baseline BMI on weight and waist circumference change. The only statistically significant interaction was with the GDQS in the weight change model. A 1-SD increase in GDQS was associated with −0.46 kg weight change among women with BMI ≥25 and with −0.21 kg weight change among women with BMI <25 (*P*-interaction = 0.004) (**Supplemental Table 1**).

## Discussion

In this longitudinal analysis of nonpregnant, nonlactating women of reproductive age, we found that participants who had the largest increase in GDQS gained less weight and waist circumference over a 2-y period. Likewise, women with the largest decrease in GDQS gained more weight and waist circumference than did women who had little change in their GDQS within a 2-y period. In addition, we observed that the GDQS had a stronger association than the MDD-W with weight gain, and a stronger association than the AHEI-2010 with waist circumference gain.

Previous studies evaluating the association between dietary quality indices and weight change reported results that are consistent with our findings. A systematic review that included 16 longitudinal studies showed that improvement in diet quality was associated with less weight gain; however, most of the studies were from high-income countries (27). Besides, Australian women participating in a randomized controlled trial who improved the Dietary Guideline Index in the intervention group had less weight gain than the control group (28). Longitudinal studies in women have found associations with less weight gain for the Alternate Mediterranean Diet, AHEI-2010, and Dietary Approaches to Stop Hypertension (in the United States) and for the ARFS (in Australia) (11, 12). Although we cannot compare between diet quality indices because of the differences in their components or cutoffs, adherence to a healthy dietary pattern was associated with less weight gain in these different populations.

We found an inverse association between change in the GDQS and waist circumference change. Few previous studies have assessed the relation between overall diet quality and waist circumference in women, and these did not find an association (29–32). These null results could be explained by the cross-sectional design or the assessment of diet only at baseline in longitudinal analysis, or by the use of a single 24-h recall, which does not capture usual intake.

We found that multiple GDQS food groups were independently associated with weight change. For example, an increase in 1 serving/d of citrus fruits, dark green leafy vegetables, cruciferous vegetables, deep orange vegetables, and fish and shellfish was associated with less weight gain. Evidence from longitudinal studies is consistent with our results. Studies in 3 cohorts in the United States found that a 1-serving/d increase of vegetables, fruits, whole grains, and nuts was associated with less weight gain long-term (33, 34). Furthermore, in our analysis, a 1-serving/d increase of red meat, refined grains, SSBs, and purchased deep fried foods was associated with more weight gain. Results from a systematic review support that greater consumption of SSBs is associated with weight gain and obesity (35). Also, prospective and cross-sectional studies have shown that meat consumption (36, 37) and fried foods intake (38) are related to weight gain.

In this analysis, we found an inverse association with waist circumference change for fish, deep orange vegetables, low fat and high fat dairy, and whole grains. These findings are consistent with previous longitudinal studies in which the higher intake of whole grains, vegetables, fruits, and high fat dairy products was associated with less waist circumference gain (39, 40). Contrary to our results, evidence from a systematic review and meta-analysis, and studies in women from a European cohort, found no association of low fat dairy and fish intakes with waist circumference change (41–43). Furthermore, we found positive associations between sweets and ice cream and purchased deep fried foods intakes and waist circumference gain. In a few previous longitudinal studies, greater consumption of sweets (44) and fried foods (45) was associated with abdominal obesity or waist circumference gain. Despite differences in the classification of sweets and fried foods between our analysis and the aforementioned cited studies, the nutritional composition of these foods, rich in added sugars, SFAs, and *trans* fat, could contribute to fat storage. In addition, in this study, *pan dulce* (sweetened breads) was included in sweets and ice cream, and *antojitos mexicanos* (Mexican deep fried foods) were classified in purchased deep fried foods. These particular foods have an important role in the Mexican diet and could explain the strong association between these 2 GDQS components and waist circumference gain.

Several mechanisms may explain the association between overall diet quality and obesity. An increase in the score of diet quality indices is related to increased consumption of healthy foods. For instance, foods with a low glycemic index have a better metabolic response, characterized by lower blood glucose concentrations, lower postprandial insulin secretion followed by a lower energy intake in subsequent meals, and satiety (46, 47). Also, healthy foods tend to have higher amounts of dietary fiber that lead to an inhibition of hunger (48). In addition, the low energy density of some plant-based foods or fish has been linked to lower weight gain. Despite the relatively high caloric density of nuts, nuts surprisingly are not associated with weight gain and are associated with reduced body weight and waist circumference (49). Unhealthy or high-glycemic foods with high energy density promote rapid digestion and absorption, related to increased insulin secretion and fat storage or body weight gain (50). Some of these mechanisms are independent of higher energy intake, and in our analysis, we adjusted for change in energy intake. In theory, energy intake is in the causal pathway from increasing dietary quality, to lowering energy intake, to gaining less weight. However, in our data, women with the largest increase in GDQS also had the largest increase in energy intake. Individuals consuming higher amounts of food overall might score higher in the GDQS because for healthy food groups a larger amount consumed would result in a higher score, although the opposite would be true for the unhealthy food groups, in that unhealthy food groups make a lower contribution to the total GDQS. For this reason, we evaluated the change in GDQS on weight and waist circumference change independently of the change in energy intake. Regardless, analyses without adjusting for change in energy intake gave slightly weaker associations, but in general the results were similar (data not shown).

We compared the performance of the GDQS with the AHEI-2010 and the MDD-W. We found that the GDQS had a stronger association than the MDD-W with weight gain, and a stronger association than the AHEI-2010 with waist circumference gain, although the differences in absolute values were small. It was not surprising that the GDQS had a stronger association with weight gain than the MDD-W; previous studies found that dietary diversity was not associated with obesity (51, 52). Dietary diversity is focused on capturing nutritional adequacy through dietary variety disregarding other nutritional characteristics (e.g., all grains are considered irrespective of whether these are refined or whole). Furthermore, the dietary diversity score does not include other unhealthy food groups that affect measures of body adiposity such as SSBs and desserts. On the other hand, it was interesting that the AHEI-2010 was outperformed by the GDQS for waist circumference, particularly considering that the GDQS aims to balance nutrient adequacy and chronic disease risk, whereas the AHEI-2010 is only focused on chronic disease risk. However, the AHEI-2010 was not developed specifically to capture obesity risk (20), and it does not include some dietary components that have been strongly associated with adiposity such as refined grains, sweets, and deep fried foods. Moreover, we found that many food groups included in the GDQS that are not considered in the AHEI-2010 were of particular importance in our population, such as fish, refined grains, sweets and ice cream, high fat dairy, and purchased deep fried foods.

A major strength of this analysis is the availability of repeated measurements of diet and anthropometric measures in a large sample of women of reproductive age from a low- and middle-income country. Slight changes in perceived weight could cause individuals to modify their dietary habits or lifestyle. For example, persons who are gaining weight might reduce their intake of SSBs and sweets or increase their consumption of vegetables, leading to reverse causation. Hence, analyses of changes in diet with concurrent changes in weight, such as the one we performed in our study, are preferable because this more closely approximates an intervention (53). Several studies have only included baseline dietary measurements and have not found an association with weight and waist circumference change (31, 54).

This study has its limitations. We had detailed lifestyle and sociodemographic information and we were able to control for many potential confounders. Nonetheless, because this is an observational study, residual confounding cannot be completely ruled out. Furthermore, anthropometric measures and dietary intakes were self-reported. However, previous validation studies in a subsample of the cohort suggested that these self-reported anthropometric measures have high validity, and diet was previously validated in Mexican women (16, 23). Also, our results might not be generalizable to all Mexican women. This cohort is composed of teachers and they have a higher education level and lower prevalence of overweight and obesity than the general population of women in Mexico. However, it is unlikely that the biological mechanisms of diet quality on weight and waist circumference change differ greatly by these characteristics. For instance, we found a strong association between changes in diet quality and weight and waist circumference regardless of baseline BMI (Supplemental Table 1). Furthermore, the distribution of important risk factors for obesity is comparable with that in the general population, and in a cross-sectional analysis of the Mexican National Nutrition Survey a negative association of the GDQS with BMI and waist circumference was reported (55). Finally, our follow-up period was short (2 y). However, results would likely be similar even with a longer follow-up. An analysis of women from the Nurses’ Health Study that examined 4-y changes in the GDQS and body weight found results in the same direction as ours using the same cutoffs for categories of change in the GDQS, but the change observed was approximately double the change we observed (26).

In conclusion, we found that improvement in diet quality over a 2-y period, reflected by an increase in the GDQS, was associated with less weight and waist circumference gain. Food groups that were key in driving this association included increased consumption of dark green leafy vegetables, cruciferous vegetables, deep orange vegetables, citrus fruits, low fat and high fat dairy, whole grains, and fish and decreased consumption of refined grains, SSBs, red meat, sweets and ice cream, and purchased deep fried foods. These findings are encouraging because they suggest that a dietary quality metric developed to capture both nutrient adequacy and chronic disease risk in the global context predicts weight and waist circumference change in Mexican women. These results also emphasize the importance of improving diet quality as part of efforts to control the global obesity pandemic.

## Supplementary Material

nxab171_Supplemental_File

## Notes

Funding for the research was provided by FHI Solutions, recipient of a Bill & Melinda Gates Foundation grant to support Intake–Center for Dietary Assessment.

Author disclosures: TTF is an Associate Editor for the Journal of Nutrition and played no role in the Journal’s evaluation of the manuscript. All other authors report no conflicts of interest.

Supplemental Table 1 is available from the “Supplementary data” link in the online posting of the article and from the same link in the online table of contents at https://academic.oup.com/jn/.

Published in a supplement to *The Journal of Nutrition*. Publication costs for this supplement were funded by the Bill & Melinda Gates Foundation in conjunction with FHI Solutions, recipient of a Bill & Melinda Gates Foundation grant to support Intake—Center for Dietary Assessment. The opinions expressed in this publication are those of the authors and are not attributable to the sponsors or the publisher, Editor, or Editorial Board of *The Journal of Nutrition*. The Supplement Coordinators for the supplement publication were Megan Deitchler, Intake—Center for Dietary Assessment at FHI Solutions, Washington, DC; and Sabri Bromage, Harvard TH Chan School of Public Health, Boston, MA.

The GDQS research initiative was launched by Intake – Center for Dietary Assessment. The research was led by Harvard T.H. Chan School of Public Health, Department of Nutrition and carried out in collaboration with researchers at the National Public Health Institute (INSP), Mexico. Funding for the research was provided by FHI Solutions, recipient of a Bill & Melinda Gates Foundation grant to support Intake – Center for Dietary Assessment.

Abbreviations used: AHEI-2010, Alternative Healthy Eating Index-2010; ARFS, Australian Recommended Food Score; GDQS, Global Diet Quality Score; GDQS+, Global Diet Quality Score positive submetric; GDQS−, Global Diet Quality Score negative submetric; MDD-W, Minimum Dietary Diversity for Women; MET, metabolic equivalent task; MTC, Mexican Teachers’ Cohort; NCD, noncommunicable disease; SEP, socioeconomic position; SSB, sugar-sweetened beverage.
