# Supplementary material for: Changes in the Global Diet Quality Score, Weight, and Waist Circumference in Mexican Women
Source: J Nutr. 2021 Oct 23;151(Suppl 2):152S–161S. doi: 10.1093/jn/nxab171 (PMC8542099; doi:10.1093/jn/nxab171)
Supplement: nxab171_Supplemental_File [file nxab171_supplemental_file.docx]

Erick Angulo *et. al.* Changes in the Global Diet Quality Score, Weight and Waist Circumference in Mexican Women

**Online Supplementary Material**

**Supplementary table 1**. Interaction between 1-SD increase in diet quality score and BMI on weight and waist circumference change in 2-y period: Mexican Teachers’ Cohort

|  | GDQS | | AHEI-2010 | | MDDW | |
| --- | --- | --- | --- | --- | --- | --- |
| Weight change, kg | | | | | | |
| BMI <25 | | -0.21 (-0.36, -0.06) | | -0.30 (-0.45, -0.14) | | -0.27 (-0.43, -0.11) |
| BMI ≥25 | | -0.46 (-0.58, -0.34) | | -0.33 (-0.45, -0.21) | | -0.25 (-0.38, -0.12) |
| *P*-interaction | | 0.0049 | | 0.6844 | | 0.8666 |
| Waist circumference change, cm | | | | | | |
| BMI <25 | | -0.52 (-0.80, -0.23) | | -0.31 (-0.60, -0.01) | | -0.37 (-0.67, -0.07) |
| BMI ≥25 | | -0.52 (-0.75, -0.29) | | -0.19 (-0.43, 0.05) | | -0.44 (-0.70, -0.19) |
| *P*-interaction | | 0.9859 | | 0.4699 | | 0.6648 |
| Values are beta coefficients (95% confidence interval).  GDQS: Global Diet Quality Score; AHEI-2010: Alternate Healthy Eating Index; MDDW: Minimum Dietary Diversity score for Women; BMI, Body Mass Index.  ^a^ Values were adjusted for baseline age (continuous); change in energy (continuous); baseline scores (continuous); state (Jalisco, Veracruz); 2006 and 2008 physical activity (low, medium, high); marital status (single, living together, married, separated, widow); education (none, ≤high school, undergraduate degree, ≥graduate degree); baseline household assets (lowest, medium, highest); health insurance (public, private, other); and changes in smoking status (baseline past smoker, stayed nonsmoker, stayed smokers, quitters, starters) and alcohol consumption (baseline nondrinkers, stayed nondrinkers, stayed drinkers, quitters, starters).  Weight change analysis: BMI <25, n=3,335; BMI ≥25, n=5,632; waist circumference change analysis: BMI <25, n=2,878; BMI ≥25, n=4,710. | | | | | | |
